# A novel prognostic model based on epithelial cell progression genes identifies OAS1 as a suppressor of bladder cancer aggressiveness

**DOI:** 10.3389/fmolb.2025.1716130

**Published:** 2026-01-08

**Authors:** Xu Su, Hui Yu, Miaoyu Zhang, Kui Zeng, Fangyang Zhong, Xuerui Chen, Yuanbiao Guo, Liangbin Lin

**Affiliations:** 1 Medical Research Center, The Third People’s Hospital of Chengdu (Affiliated Hospital of Southwest Jiaotong University), College of Medicine, Southwest Jiaotong University, Chengdu, Sichuan, China; 2 Department of Urology, The Affiliated Hospital of Southwest Jiaotong University, The Third People’s Hospital of Chengdu, Chengdu, China; 3 Obesity and Metabolism Medicine-Engineering Integration Laboratory, Department of General Surgery, The Affiliated Hospital of Southwest Jiaotong University, The Third People’s Hospital of Chengdu, Chengdu, China; 4 The Center of Gastrointestinal and Minimally Invasive Surgery, Department of General Surgery, The Affiliated Hospital of Southwest Jiaotong University, The Third People’s Hospital of Chengdu, Chengdu, China

**Keywords:** bladder cancer, OAS1, prognostic model, single-cell RNA sequencing, tumour-progressing epithelial cells

## Abstract

**Background:**

Bladder cancer (BLCA) is a highly heterogeneous malignancy with an unpredictable prognosis. Tumour progression is closely linked to the complex tumour microenvironment (TME), particularly the role of epithelial cells. This study aims to identify key epithelial cell-derived signature genes driving tumour progression, construct a reliable prognostic model, and further explore the biological functions of a pivotal gene, *OAS1*, in BLCA.

**Methods:**

Single-cell RNA sequencing (scRNA-seq) data from public cohorts were analyzed to identify epithelial cell subpopulations and delineate their malignant progression trajectory. Genes significantly associated with this progression were identified through pseudotime analysis. Bulk RNA-seq and clinical data from The Cancer Genome Atlas (TCGA) BLCA cohort were utilized for least absolute shrinkage and selection operator (LASSO) Cox regression to build a prognostic risk model. The model’s predictive efficacy was validated in an independent Gene Expression Omnibus (GEO) cohort. Furthermore, *in vitro* experiments including CCK-8, transwell, and wound healing assays were conducted to investigate the impact of OAS1 on the proliferation, migration, and invasion capabilities of BLCA cells.

**Results:**

scRNA-seq analysis revealed a distinct epithelial cell subpopulation with high tumor-suppressive activity. A four-gene signature associated with tumor progression was successfully constructed into a prognostic model. Patients in the high-risk group exhibited significantly poorer overall survival in both the TCGA and validation cohorts. Multivariate Cox analysis confirmed the model as an independent prognostic factor. The risk score was significantly correlated with immune infiltration patterns and response to immunotherapy. Among the signature genes, *OAS1* was identified as a critical factor. *In vitro* functional experiments demonstrated that knockdown of *OAS1* markedly promoted the proliferation, migration, and invasion of BLCA cells.

**Conclusion:**

We established a novel prognostic model for BLCA based on epithelial cell tumor progression-associated genes, which serves as a robust predictor for patient outcomes and immunotherapeutic responsiveness. Our findings further highlight *OAS1* as a key gene that suppresses the aggressive phenotypes of BLCA cells, suggesting it is a potential therapeutic target. This study provides valuable insights for precise prognosis and treatment stratification of BLCA patients.

## Introduction

BLCA is the fourth most common cancer among men (based on 2024 statistics), accounting for 6% of projected new cancer cases and 4% of cancer-related deaths (Siegel et al., 2024), which is a common malignancy initiated by abnormal proliferation of urothelial cells on the inner surface of the bladder (Zheng et al., 2021). About 90% of bladder cancer cases are urothelial carcinomas, and most of the rest are squamous-cell carcinomas, adenocarcinomas, or neuroendocrine carcinomas. Carcinoma *in situ* accounts for about 10% of non-muscle invasive bladder cancer (Mohanty et al., 2023; Flaig et al., 2022; Dyrskjøt et al., 2023; Kamat et al., 2016; Konieczkowski et al., 2021; Lobo et al., 2022). As one of the top 10 most common cancers in the world, bladder cancer poses a significant burden to public health (Dobruch et al., 2016; Jiménez-Guerrero et al., 2021). Patients with bladder cancer can be divided into non-muscle-invasive bladder cancer (NMIBC) and muscle-invasive bladder cancer (MIBC) according to whether the tumor invades the muscle layer of the bladder wall. Non-muscle invasive bladder cancer accounts for 70%–80% of the cases; the prognosis is good, but the recurrence rate is high. Muscle-invasive bladder cancer accounts for 20%–30% of cases, with aggressive tumor behavior, metastatic potential, and poor overall survival (Mertens et al., 2014). The clinical importance of BLCA lies not only in its prevalence but also in the substantial costs associated with its long-term management and surveillance. Despite advances in surgical techniques and adjuvant therapies, patient outcomes, particularly for those with advanced or metastatic disease, remain suboptimal, underscoring the urgent need for more effective prognostic tools and therapeutic strategies.

A major challenge in improving BLCA management stems from its profound molecular heterogeneity (Ding et al., 2022; Li et al., 2023; Li and Fang, 2024; Qian et al., 2024). This diversity contributes to considerable variations in tumor behavior, treatment response, and overall survival, making accurate diagnosis, risk stratification, and personalized treatment planning exceptionally difficult. Consequently, there is a growing emphasis on identifying robust molecular biomarkers that can capture this heterogeneity and provide more precise prognostic information.

Among the various molecular players, genes associated with the urothelium, the epithelial lining of the urinary bladder, are gaining attention for their pivotal roles in cancer development. These genes are critically involved in maintaining cellular energy homeostasis, regulating intricate signal transduction pathways, and modulating the TME. Their dysregulation can fuel tumour progression, influence immune cell infiltration, and potentially shape response to therapies, including immunotherapy. Notably, the *OAS1* (2′-5′-oligoadenylate synthetase 1) gene, a key component of the innate immune response against viral infections, has recently been implicated in various cancers (Lu et al., 2022; Kondratova et al., 2020; Zhang and Yu, 2020; Yu et al., 2018). It is a multifunctional effector protein that, as a member of the OAS protein family, participates in a series of cellular biological processes (Conn et al., 2020). A study employed bioinformatics methods to explore the role of OAS1 in pan-cancer (Jiang et al., 2023). The expression level of OAS1 varies across different tumor types and plays distinct roles in different cancers. Notably, the expression level of OAS1 in normal BLCA tissues was significantly higher than that in cancerous tissues, suggesting that the OAS1 gene may function as a tumor suppressor in BLCA. Its specific role in the context of tumour-progressing epithelial cells (TPECs) and its impact on BLCA progression, however, remains inadequately explored.

Therefore, the primary objective of this study is to leverage TPECs-related genes, with a specific focus on OAS1, to construct a novel prognostic model for bladder cancer. We aim to systematically investigate the expression pattern and biological functions of OAS1, assess its association with patient prognosis, and elucidate its relationship with the tumor immune microenvironment and potential response to immunotherapy. This research aims to provide a valuable molecular tool for refining risk assessment and uncovering new insights into the biological mechanisms underlying bladder cancer progression.

## Materials and methods

### Single-cell RNA sequencing data acquisition and preprocessing

The single-cell RNA sequencing (scRNA-seq) dataset used in this study was derived from a previously published Study (Mohanty et al., 2023). Specifically, nine BLCA patients who underwent bladder tumor resection (TURBT) or cystectomy were included. There were eight men and one woman, with five having NMIBC and four having MIBC. Only eight patients had paired tumor samples collected. Single-cell suspensions were prepared and sequenced using a droplet-based single-cell sequencing platform (10X Genomics) following the manufacturer’s standard protocol (Flaig et al., 2022). Sequencing data underwent strict quality control, with cells containing more than 15% (This threshold is based on common practice and is used to exclude low-quality or apoptotic cells) mitochondrial gene content excluded to remove dead or dying cells; Cells with too low (<200) and too high (>5,000) gene numbers were filtered to reflect the complexity of the transcriptome; cells with too low (<500) and too high (>3,000) UMI were excluded; the hemoglobin gene expression ratio was set below 5%; The ratio of ribosomal gene expression was set above 60 percent. Data normalisation, dimensionality reduction, and cell clustering analysis were performed using the Seurat R package. Cell type annotation was based on classical gene markers established in the published literature. The final dataset integrates high-quality single-cell transcriptome profiles to comprehensively capture distinct immune cells, stromal cells, and epithelial cell populations in the urethral microenvironment of BLCA. Single-cell RNA sequencing (scRNAseq) data have been deposited in the GEO (accession number: GSE267718) and are publicly available.

### Bulk RNA-seq and clinical data acquisition from TCGA and E-MTAB-4321-ArrayExpress

Based on the studies mentioned above, we extracted TPECs for analysis. We then obtained tissue expression data and clinical follow-up information from bladder cancer patients in TCGA for validation. The data preprocessing involved three main steps: (1) removing specimens lacking relevant clinical records, (2) converting all identifiers to standardised gene nomenclature, and (3) selecting the highest expression value when multiple entries existed for the same gene. After preprocessing, we downloaded 411 samples from TCGA-BLCA, and 476 samples were obtained from E-MTAB-4321-ArrayExpress (Supplementary Table S1).

### The consensus ClusterPlus algorithm was used to identify molecular subtypes

The TCGA expression profiles were filtered to retain TPEC-related genes with reliable expression, defined as genes exhibiting expression levels greater than 1 in at least 50% of the samples. Subsequently, univariate Cox regression analysis was conducted to identify epithelial-related genes significantly associated with prognosis (P < 0.01). Using the expression profiles of the seven identified prognostic epithelial-related genes, non-negative matrix factorisation (NMF) clustering was employed to classify TCGA samples into distinct molecular subtypes. NMF analysis was performed using the Brunet algorithm in the R package NMF (v0.26), with cluster ranks (k = 2–10) and 50 iterations to evaluate stability. The optimal number of clusters was determined based on cophenetic correlation coefficients and residual sum of squares (RSS), with k = 2 selected due to the highest cophenetic correlation coefficient (≥0.99) and a significant reduction in RSS. Consensus matrices were generated to visually assess and validate the stability of the clusters.

### Lasso Cox regression analysis

To optimize the prognostic model, LASSO regression was employed for feature selection among 1,054 differentially expressed genes. This method utilizes L1 regularization to address multicollinearity while simultaneously performing variable selection and parameter estimation, thereby enhancing model interpretability. The procedure comprised: (1) construction of a regularized Cox model using the GLMNET package, (2) coefficient path analysis, (3) determination of the optimal λ value via five-fold cross-validation, the λ value with the smallest cross-validation error (or the smallest λ value within one standard error of the error) is selected and (4) confidence interval evaluation to identify robust gene subsets. Model optimization was guided by three criteria: (1) cross-validation error minimization, (2) optimal balance between model simplicity and predictive power, and (3) biological relevance of the markers. This process reduced the original gene set to compact, informative feature combinations while preserving prognostic performance. The LASSO method was particularly effective for analyzing genomic data, eliminating redundant variables, and generating sparse solutions amenable to clinical translation, thereby bridging genomic analysis with clinical applications.

### Lentiviral production and infection

Human embryonic kidney cell line HEK293 and human bladder cancer cell lines T24 were obtained from the Cell Bank of Type Culture Collection of Chinese Academy of Sciences (Shanghai, China; http://www.cellbank.org.cn/). HEK293 cells were maintained in high-glucose DMEM supplemented with 10% fetal bovine serum (FBS) and 1% penicillin-streptomycin. T24 cells were cultured in RPMI-1640 medium containing 10% FBS and 1% penicillin-streptomycin. All cell lines were incubated at 37 °C in a humidified atmosphere with 5% CO_2_ (Thermo Scientific, Forma 3,111). Cells were passaged every 2 days at 70%–80% confluence using 0.25% trypsin-EDTA (Gibco, 25200056) for detachment. All experiments were performed using cells within passage 20.

Short hairpin RNA against negative control (NC-shRNA) and OAS1(OAS1-shRNA) were synthesised by Tsingke (Beijing, China). The sequences are detailed in Table 1.

**TABLE 1:** OAS1 plasmid sequences.

Plasmids	Sequences
sh*OAS1*-1	GATCGGCTGAATTACCCATGCTTTAACTCGAGTTAAAGC ATGGGTAATTCAGCTTTTTT
sh*OAS1*-2	GATCGGGGAGAGTTCATCCAGGAAATCTCGAGATTTCCT GGATGAACTCTCCCTTTTTT
sh*OAS1*-3	GATCGCAGTTGACTGGCGGCTATAAACTCGAGTTTATAG CCGCCAGTCAACTGTTTTTT
sh*OAS1*-4	GATCGATCTACTGGACAAAGTATTATCTCGAGATAATACT TTGTCCAGTAGATTTTTTT

Lentiviral particles were generated through calcium phosphate transfection. HEK293 cells were plated in 10 cm dishes and co-transfected at a 1.7:1:1.3 ratio with shRNA constructs, packaging plasmid psPAX2, and envelope plasmid pMD2.G (encoding VSVG) using calcium phosphate precipitation. Following 6–8 h of incubation, the medium was replaced with fresh complete medium. Viral supernatants were harvested 48 h post-transfection and clarified through centrifugation (2,000 rpm, 5 min) to remove cellular debris. Target cells were transduced with lentiviral particles (3 μg/mL polybrene) for 24–48 h, with stable transductions selected using puromycin where appropriate.

### Quantitative reverse transcription PCR (qRT-PCR)

Total RNA was extracted using the RNeasy Mini Kit (Qiagen, 74,101) following the manufacturer’s protocol. cDNA was synthesised from 1 μg of total RNA, followed by qRT-PCR with 500 ng of cDNA in 20 μL reactions. Reactions were performed using iQ™ SYBR® Green Supermix (Bio-Rad) with 500 ng of cDNA in 20 μL reaction volumes. We used two forward primers and two reverse primers. Primer sequences and annealing temperatures are provided in Table 2. Gene expression was normalised to the *ACTB* reference gene.

**TABLE 2:** qPCR primer information.

Name of the primers	Primer sequences	Annealing temperature
*OAS1*-1 F	TTTCCGCATGCAAATCAACC	57.92
*OAS1*-1 R	GATCGGCCTCTGAGGGT	57.55
*OAS1*-2 F	AGTTTGAGGTCCAGGCTCC	58.93
*OAS1*-2 R	GGGGTTAGGTTTATAGCCGC	58.11
h*ACTB* F	GCCTCGCCTTTGCCGAT	60.00
h*ACTB* R	CGCGGCGATATCATCATCC	59.00

### Cell proliferation assay

After cells were transfected with *OAS1*-siRNA, Cell proliferation assays were performed using T24 cells in 96-well plates. Cells were seeded in 100 μL of 10% FBS RPMI 1640 at a density of 2000 cells/well, and cells were cultured for the indicated days. For the Cell Counting Kit-8 (CCK-8) assay, the original medium in each group was replaced with 10 μL of CCK-8 solution (diluted in 100 μL of complete medium) according to the protocol provided by Biosharp. After incubation for an additional 0.5, 1, and 2 h in the dark at 37 °C, viable cells were detected by measuring absorbance at a wavelength of 450 nm.

### Wound healing assay

Cells were plated in 6-well plates and cultured to ∼90% confluence before wounding. Monolayers were wounded using sterile 200 μL pipette tips. Following PBS washes to remove debris, cells were maintained in serum-free medium. Wound areas were imaged at 0, 12, and 24 h post-wounding using phase-contrast microscopy. Migration rates were calculated as: % closure = [1 - (wound width at Tx/wound width at T0)] × 100. Migration kinetics were quantified at 12 and 24 h post-wounding.

### Transwell assay

The invasive ability of cells was quantitatively assessed using a modified Boyden chamber assay with Matrigel-coated transwell inserts. After genetic manipulation, approximately 1.5 × 10^5 cells in serum-free medium were carefully seeded into the upper chamber of each Transwell. The lower chamber was filled with complete medium containing 10% fetal bovine serum, serving as a strong chemoattractant to promote cell migration through the extracellular matrix barrier. After a 24-h incubation at 37 °C in a humidified 5% CO_2_ atmosphere, non-invading cells remaining on the upper surface of the membrane were removed mechanically with cotton swabs. Cells that had migrated through the Matrigel and attached to the lower membrane surface were fixed with 4% paraformaldehyde for 15 min at room temperature, then stained with 0.1% crystal violet solution for 30 min. Quantitative analysis involved counting stained cells in five randomly selected fields per membrane under an inverted phase-contrast microscope (Leica, ×10 magnification). All experiments were performed in triplicate and repeated three times independently to ensure statistical reliability.

## Results

### Single-cell profiling reveals distinct cellular landscapes and TPECs gene signatures in MIBC vs. NMIBC

**FIGURE 1:**
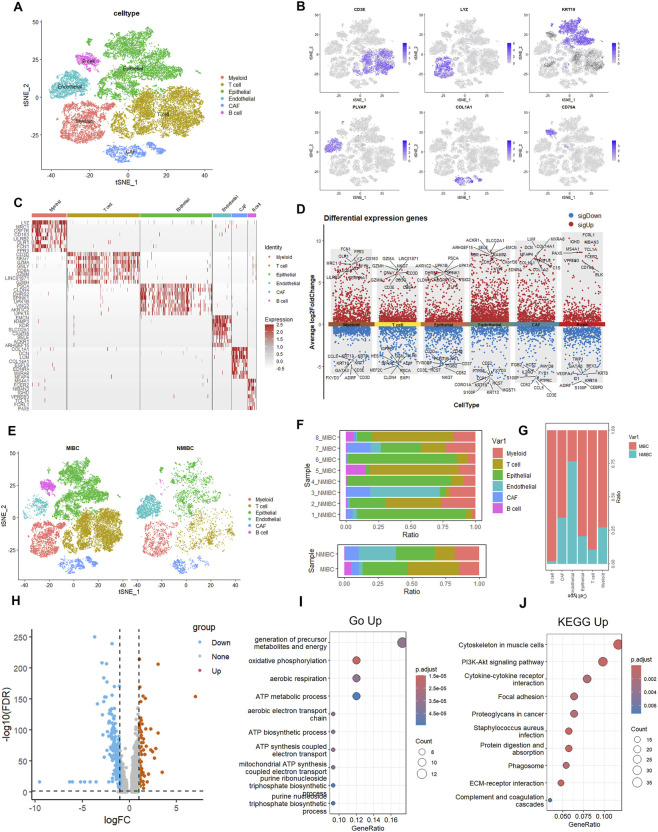
Single-cell RNA-sequencing analysis of cellular composition and gene expression patterns. **(A)** t-SNE plot shows the clustering of different cell types, including Myeloid, T cell, Epithelial, Endothelial, CAP, and B cell. **(B)** Feature plots illustrate the expression of specific marker genes (CD68, LYZ, KRT19, PDL1, COL4A1, CD34) across cells. **(C)** Heatmap depicting the expression of canonical marker genes across major cell types identified by single-cell RNA sequencing (scRNA-seq) in bladder cancer samples. **(D)** Volcano plot of differentially expressed genes between cell types, with red dots representing significantly up-regulated genes and blue dots representing significantly downregulated genes. **(E)** t - SNE plots of cells from MIBC and NMIBC samples, colored by cell type. Among a total of 30,428 cells, the cellular composition of the MIBC sample (23,274 cells) includes myeloid cells (3,461), T cells (8,894), epithelial cells (7,812), endothelial cells (593), CAFs (1,388), and B cells (1,126); the NMIBC sample (7,154 cells) is composed of myeloid cells (1,301), T cells (1,072), epithelial cells (2,037), endothelial cells (1,984), CAFs (737), and B cells (23). **(F)** Stacked bar plots showing the proportion of each cell type in different MIBC (7_MIBC, 5_MIBC, 3_MIBC, 2_MIBC, 1_MIBC) and NMIBC samples. **(G)** Bar plot comparing the proportion of Myeloid cells between MIBC and NMIBC groups. **(H)** Volcano plot of differentially expressed genes between MIBC and NMIBC groups, with blue dots for down-regulated, red dots for up-regulated, and grey dots for non-significant genes. **(I)** GO enrichment analysis of up-regulated genes in MIBC compared to NMIBC, showing the top enriched biological processes. **(J)** KEGG pathway enrichment analysis of up-regulated genes in MIBC compared to NMIBC, highlighting the significantly enriched pathways.

We analyzed single-cell data from 4 MIBC and 4 NMIBC tumors each. Significant cellular populations were systematically classified, including myeloid, T, epithelial, endothelial, and B cell lineages (Figure 1A). Cellular identities were confirmed using established markers: *LY6A* (myeloid), *CD3E* (T cells), *KRT19* (epithelial), *PLVAP* (endothelial), *COL1A1* (stromal), and *CD79A* (B cells) (Figures 1B–D). Cellular distribution patterns were compared between muscle-invasive (MIBC) and non-muscle-invasive (NMIBC) bladder cancer specimens (Figure 1E). Significant compositional differences were observed between these clinical subtypes. The MIBC cohort (n = 23,274 cells) was primarily composed of T lymphocytes, with epithelial cells as the secondary population. Conversely, NMIBC specimens (n = 7,154 cells) showed an opposite pattern, with epithelial cells dominating, followed by endothelial cells (Figure 1F). Quantitative analysis indicated a significantly higher proportion of epithelial cells in MIBC compared to NMIBC (Figure 1G).

Consequently, differential gene expression analysis was performed on epithelial compartments comparing MIBC and NMIBC. Genes were categorized as upregulated (logFC >1, p < 0.05) or downregulated (logFC < −1, p < 0.05). A total of 996 genes were upregulated in advanced bladder cancer, providing a basis for subsequent functional enrichment analysis (Figures 1H–J). GO analysis showed significant enrichment for mitochondrial oxidative phosphorylation, ATP metabolic processes, and aerobic respiration. Upregulated genes are primarily associated with electron transport chain components and purine nucleotide biosynthesis (Figure 1I). KEGG pathway analysis identified two main theme clusters: 1) neurodegenerative pathways (Parkinson’s, Alzheimer’s, and Huntington’s diseases), and 2) oxidative stress networks (ROS-mediated carcinogenesis, NAFLD, and redox homeostasis) (Figure 1J).

The consistent identification of oxidative phosphorylation in both analyses emphasizes metabolic dysregulation as a key feature. The co-enrichment indicates shared pathophysiology involving mitochondrial dysfunction, chronic oxidative stress, and bioenergetic imbalance. These results suggest that ROS-mediated oxidative stress could be a driving force behind both cancer development and metabolic disruption.

### TPECs related molecular subtype identification based on an NMF algorithm

**FIGURE 2:**
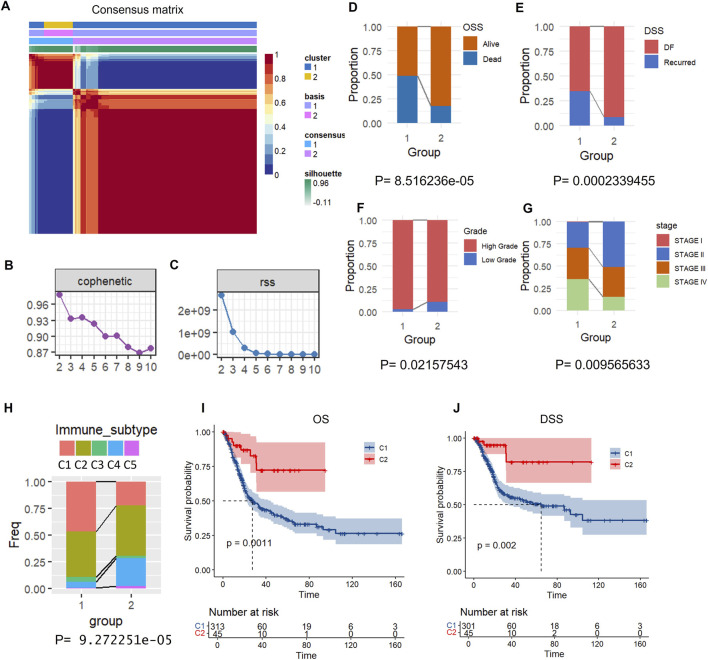
Molecular subtyping and prognostic analysis of bladder cancer based on NMF clustering. **(A)** Consensus matrix heatmap for clustering samples into two groups (Cluster 1 and Cluster 2), with colour intensity representing consensus value. **(B)** Cophenetic distributions of rank 2–10, where COPHENOTYPIC correlations are derived from the concordance matrix proposed by Brunet et al., reflect the clustering stability obtained by non-negative matrix factorisation (NMF). The values range from 0 to 1, with higher values indicating more stable clusters. **(C)** RSS distribution, rank = 2–10. RSS is the sum of the residual squares, representing the clustering performance of the model. The smaller the value is, the better the clustering effect of the model is. **(D–G)** Comparing the distribution of the two molecular subtypes in different clinical characteristics in the TCGA dataset. **(H)** Comparison of the distribution of molecular immune subtypes in two subtypes. **(I)** Overall survival (OS) prognostic survival curve of molecular subtypes. **(J)** Prognostic Survival Curves of DSS by molecular subtype.

Expression profiles of 996 TPECs-related genes were extracted from the TCGA-BLCA dataset. Univariate Cox regression (using the R survival package) identified genes significantly associated with clinical outcomes (p < 0.001), leading to the selection of seven prognostic genes (Supplementary Table S2). NMF clustering grouped BLCA cases based on these seven markers. NMF analysis revealed two molecular subtypes (C1/C2; Figures 2A–C). Cluster separation was confirmed through consensus heatmaps (showing intra-cluster similarity), survival curve analysis (log-rank p < 0.05), and PCA visualization (highlighting expression differences). This classification provides insight into epithelial heterogeneity in BLCA progression.

Comparative analysis of clinical characteristics between the two molecular subtypes revealed significant differences in key prognostic parameters. First, Kaplan-Meier survival analysis showed a markedly worse overall survival outcome in subtype C1 compared to the other group (log-rank P < 0.05, Figure 2D). Second, the tumor recurrence rate was significantly higher in subtype C1 (χ^2^ test, P < 0.01, Figure 2E), indicating that this subgroup may have a more aggressive disease phenotype. C1 subtype has more patients with high grade and high stage (Figures 2F,G). To further explore the relationship between molecular subtypes and the tumor immune microenvironment, we analyzed the distribution differences of C1 and C2 subtypes across established immune subtypes. The proportion of immunosuppressive subtypes (e.g., C1/C2 immune subtypes) was significantly higher in the C1 subtype, while the C2 subtype was more enriched in immune-inflamed subtypes (e.g., C3/C4 immune subtypes) (Figure 2H). This finding suggests that the molecular classification based on TPECs-related genes is closely associated with the characteristics of the tumor immune microenvironment. These findings collectively suggest that our molecular classification system effectively stratifies patients based on clinically relevant outcomes. C1 patients had significantly shorter OS and DSS compared to C2 (Figures 2I,J; log-rank p < 0.05).

### The poor-prognosis subtype is associated with a higher immune score

To thoroughly assess the differences in the tumour immune microenvironment between the two molecular subtypes (C1 and C2), we conducted a systematic immune profiling of the TCGA dataset using several established algorithms. The analysis included: stromal, immune, and ESTIMATE scores calculated with the ESTIMATE package (v1.0.13), which provide overall assessments of non-tumour cellular components; MCP-counter for measuring 10 distinct immune cell populations; and CIBERSORT for deconvolution analysis of 22 immune cell subsets. This multi-platform approach enabled a comprehensive characterisation of the immune landscape associated with each molecular subtype.

**FIGURE 3:**
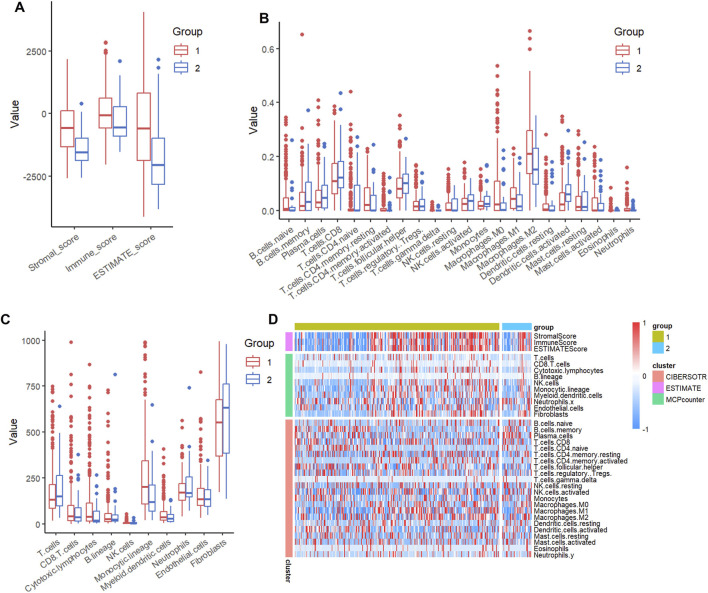
Comparative analysis of the immune score of molecular subtypes of bladder cancer using multiple immune software in the TCGA dataset. Comparison of three immune scores among molecular subtypes in the TCGA dataset. **(A)** Estimate immune score comparison between molecular subtypes in the TCGA dataset. **(B)** Comparison of CIBERSOTR immune scores among molecular subtypes in the TCGA dataset. **(C)** Comparison of MCPcounter immune scores among molecular subtypes in the TCGA dataset. **(D)** Heatmap of immune score comparison among molecular subtypes in the TCGA dataset by three immune software.

Comparative analysis revealed significant differences in immune microenvironment characteristics between the two molecular subtypes. As shown in Figures 3A–C, the C1 subtype exhibited consistently higher stromal, immune, and ESTIMATE scores across all three analytical platforms compared to the C2 subtype (all P < 0.05). These findings were further supported by unsupervised clustering analysis of immune scores, which demonstrated clear separation between the subtypes (Figure 3D), suggesting distinct immunological profiles associated with each molecular classification. The robust concordance across multiple scoring methods strengthens the validity of these observed immune differences.

### Identification of differentially expressed genes between subtypes

**FIGURE 4:**
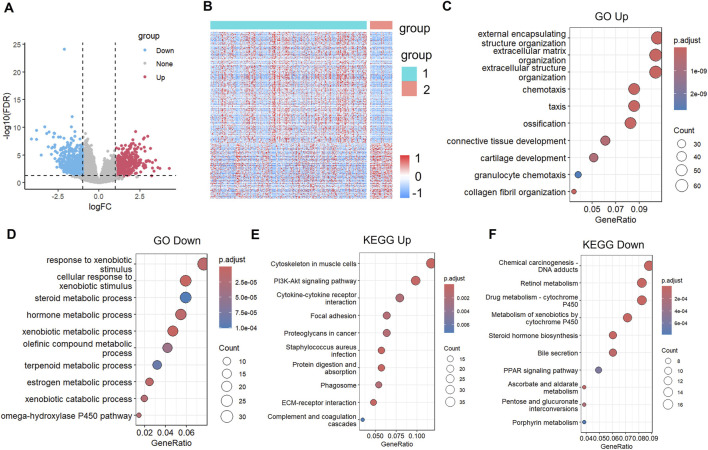
Differential gene expression analysis and functional annotation of molecular subtypes of bladder cancer. **(A)** Volcano plot of differentially expressed genes in C1 and C2 groups. **(B)** Heatmap of differentially expressed genes in two groups. **(C)** Biological process (BP) annotation map of differentially upregulated genes in molecular subtypes. **(D)** (Biological process) BP annotation map of differentially downregulated genes in molecular subtypes. **(E)** KEGG annotation map of differentially upregulated genes in molecular subtypes (quoted from www.KEGG.jp/KEGG/kegg1.html). **(F)** KEGG annotation of differentially downregulated genes in molecular subtypes.

Differential gene expression analysis between the C1 and C2 subtypes was carried out using the limma package with strict criteria (|log2FC| > 1 and FDR <0.01). This analysis identified 1,208 significantly dysregulated genes, including 703 up-regulated and 505 down-egulated genes in C1 compared to C2 (Supplementary Table S3). Notably, most of the differentially expressed genes (DEGs) showed higher expression in the C1 subtype (Figure 4A). Unsupervised hierarchical clustering of these DEGs demonstrated clear expression patterns that separated the two molecular subtypes, further confirming their molecular differences (Figure 4B). The complete list of DEGs is available in Supplementary Table S3.

Functional enrichment analysis of the 703 up-regulated genes in the BLCA C1 subtype was performed using the Goplot R package. The results revealed significant enrichment in several biologically relevant pathways (Figure 4C), notably including external encapsulating structure organisation, extracellular matrix organisation, and extracellular structure organisation. These findings suggest that the C1 subtype exhibits distinct metabolic characteristics, particularly in the processing of xenobiotics and steroid hormones, which may contribute to its aggressive clinical behaviour. GO functional enrichment analysis was performed on the 505 downregulated genes (Figure 4D). The results revealed that these genes were significantly enriched in pathways related to the “response to xenobiotic stimulus, steroid metabolic process, hormone metabolic process, and xenobiotic metabolic process”. This finding suggests that the C1 subtype may exhibit reduced activity or functional dysregulation in biological processes such as xenobiotic detoxification, steroid metabolism, and hormone metabolism. KEGG pathway enrichment analysis of the upregulated genes in the C1 subtype was performed using the Goplot R package. The results revealed significant enrichment in multiple pathways (Figure 4E), including “Cytoskeleton in muscle cells, PI3K-Akt signaling pathway, cytokine-cytokine receptor interaction, focal adhesion, proteoglycans in cancer, Staphylococcus aureus infection, protein digestion and absorption, phagosome, ECM-receptor interaction, and complement and coagulation cascades”. These pathways are primarily associated with extracellular matrix organization, cell adhesion, immune response, and metabolic regulation. These findings further support the active state of the C1 subtype in terms of cellular structural reorganization, signal transduction, and microenvironment adaptation, which may contribute to its aggressive phenotype and poor prognosis. Further integrated KEGG pathway enrichment analysis was performed on the differentially expressed genes between the C1 and C2 subtypes (Figure 4F). The results showed that the downregulated genes were significantly enriched in a series of pathways closely related to metabolism and carcinogenic processes, including chemical carcinogenesis-DNA adduct formation, retinol metabolism, drug metabolism-cytochrome P450, metabolism of xenobiotics by cytochrome P450, steroid hormone biosynthesis, and the PPAR signaling pathway, among others. These findings collectively suggest that, compared to the C2 subtype, the C1 subtype exhibits widespread reduced activity or functional dysregulation in the metabolic transformation, detoxification, and energy balance regulation of various endogenous and exogenous substances (such as drugs, xenobiotics, steroids, and lipids). This systemic alteration of the metabolic network, particularly the dysregulation of pathways related to carcinogen activation, hormonal homeostasis, and energy sensing, may underlie the unique metabolic remodeling of the C1 subtype and be closely associated with its more aggressive clinical behavior.

### Construction of predictive risk models

**FIGURE 5:**
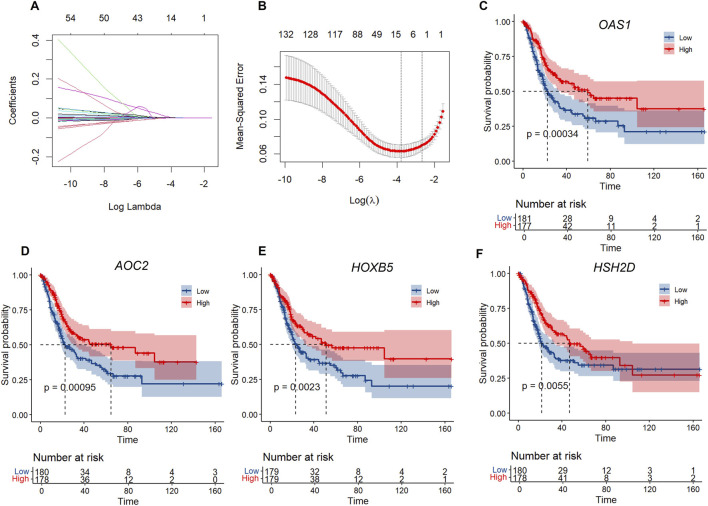
LASSO regression and survival analysis of key genes. **(A)** Independent variable locus: horizontal axis (logarithm of dependent variable) and vertical axis (coefficient of independent variable. **(B)** Confidence intervals for each λ. **(C–F)** KM curves of four genes in TCGA.

To further develop the risk score model, we first identified DEGs between C1 and C2, resulting in a total of 1054 DEGs. Univariate Cox regression analysis of survival data showed that 132 genes were significantly associated with prognosis (using p < 0.01 as the screening threshold) (Supplementary Table S4). However, this large number of genes is impractical for clinical testing. Therefore, we applied Lasso regression to reduce the gene list further. Lasso Cox regression analysis was conducted with the R package glmnet to examine the trajectories of the independent variables (Figure 5A). The results indicate that the number of independent variable coefficients increases as λ increases. Cross-validation was used to assess confidence intervals at each λ value. As shown in Figure 5B, the model performs well at λ = 0.02279. At this point, nine genes were selected as the target genes.

A stepwise regression based on the Akaike Information Criterion (AIC) (variable selection was performed using the backward selection method) is then used to assess the statistical fit of the model and its parameters. In the MASS package, the AIC method begins with a complex model and simplifies it by removing variables, resulting in a more refined model with fewer parameters. We used this algorithm to narrow down the nine genes mentioned above to four: *OAS1*, *AOC2*, *HOXB5*, and *HSH2D*. The prognostic Kaplan-Meier (KM) curves for these four genes were subsequently plotted. As shown in Figures 5C–F, these four genes were positively associated with survival in the TCGA training set samples (p < 0.05).

**FIGURE 6:**
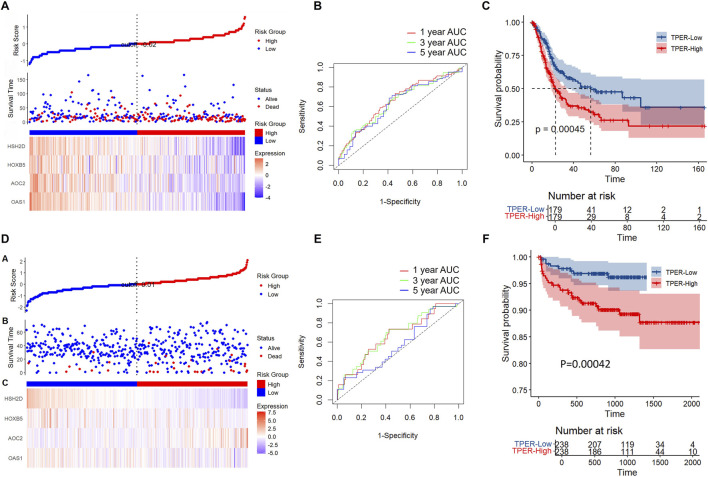
RiskScore model and survival analysis. **(A)** Risk score distribution, survival status, and gene expression heatmap for OAS1, HOXB5, AOC2, and HSH2D in the cohort, with samples divided into high - and low-risk groups. **(B)** Time-dependent ROC curves for 1-year, 3-year, and 5-year overall survival (OS) in the cohort, showing the area under the curve (AUC) for the risk score model. **(C)** Kaplan - Meier curve for OS in the training cohort stratified by the risk score (TPER-Low vs. TPER-High), with a P-value of 0.00045. **(D)** Risk score distribution, survival status, and gene expression heatmap for OAS1, HOXB5, AOC2, and HSH2D in the validation cohort, with samples divided into high - and low-risk groups. **(E)** Time-dependent ROC curves for 1-year, 3-year, and 5-year OS in the validation cohort, showing the AUC for the risk score model. **(F)** Kaplan - Meier curve for OS in the validation cohort stratified by the risk score (TPER-Low vs. TPER-High), with a P-value of 0.00042.

Using the GGRISK package, we calculated a risk score for each sample based on the expression levels of the four genes, with a higher risk score indicating a worse prognosis (Figure 6). R-wrapper time ROC analysis of prognostic stratification of the risk score at 1, 3, and 5 years showed an area under the curve (AUC) higher than 0.6 for this model (Figure 6B). Finally, the sample was divided into a high-risk score group and a low-risk score group, and KM curves were plotted (Figure 6C). The prognosis of the high-risk score group was significantly worse than that of the low-risk score group, suggesting the clinical value of our risk score.

### Verifying the robustness of the 4-gene signature in external datasets

To further clarify the generalizability of the four gene signatures we screened, we selected the E-MTAB-4321-ArrayExpress dataset to validate the risk score. We use the same model and have the same coefficients. The risk score was calculated separately for each sample based on its mRNA level, and then the risk score distribution was plotted.

Consistent with our previous findings, the dataset showed that samples with a high RiskScore had a poor prognosis (Figure 6D). The prognostic prediction accuracy of RiskScore at 1, 1, 3, and 5 years was evaluated using the R package timeROC (Figure 6E), with an area under the curve (AUC) above 0.55. The KM curve also indicated that the group with a high RiskScore had a significantly worse prognosis than the group with a low RiskScore (Figure 6F).

### Risk model correlation analysis with clinical characteristics and pathways

Next, we employed gene set enrichment analysis (GSEA) to investigate the relationship between biological functions and risk scores using gene expression profiling. First, we calculated the single-sample gene set enrichment analysis (SSGSEA) scores for each sample in the KEGG pathway (KEGG). We then examined the correlation between the KEGG pathway and the risk score.

**FIGURE 7:**
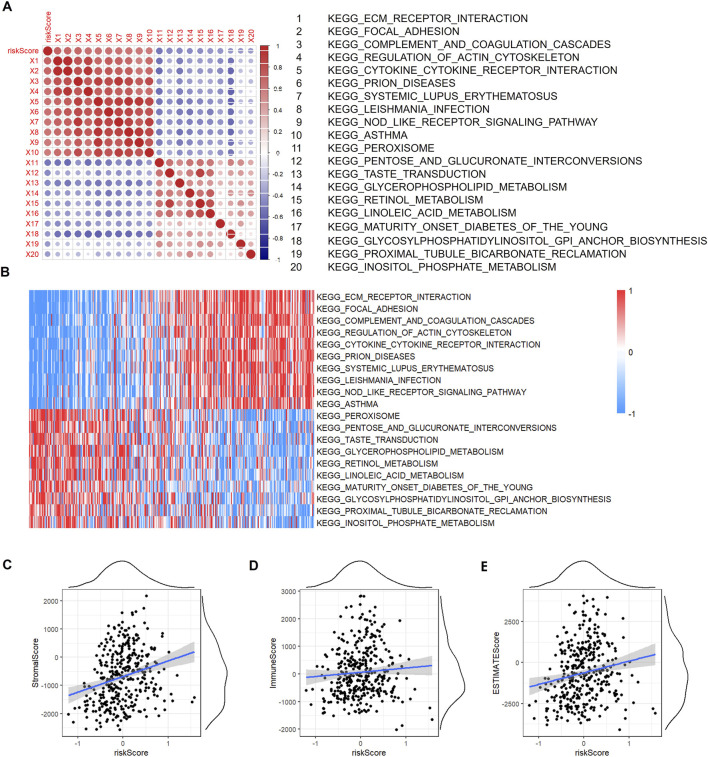
The correlation between SSGSEA and riskcore. **(A)** Correlation between SSGSEA KEGG signalling pathway score and Riskscore. **(B)** Heat map of SSGSEA KEGG signalling pathway. **(C)** Correlation between StromalScore and RiskScore. **(D)** Correlation between ImmuneScore and RiskScore. **(E)** Correlation between ESTIMATEScore and RiskScore.

Figures 7A,B show the KEGG pathways, including EGG_ECM_RECEPTOR_INTERACTION, KEGG_FOCAL_ADHESION, and KEGG_COMPLEMENT_AND_COAGULATION_CASCADES, which are positively correlated with risk scores, and the KEGG pathways, including KEGG_INOSITOL_PHOSPHATE_METABOLISM, KEGG_PROXIMAL_TUBULE_BICARBONATE_RECLAMATION, and KEGG_GLYCOSYLPHOSPHATIDYLINOSITOL_GPI_ANCHOR_BIOSTYNHESIS, which are negatively correlated with risk scores.

The correlation between the risk score and the immune score was analysed using the R package. As shown in Figures 7C–E, the risk score was positively correlated with stromal score, immune score, and estimates score (p < 0.05).

**FIGURE 8:**
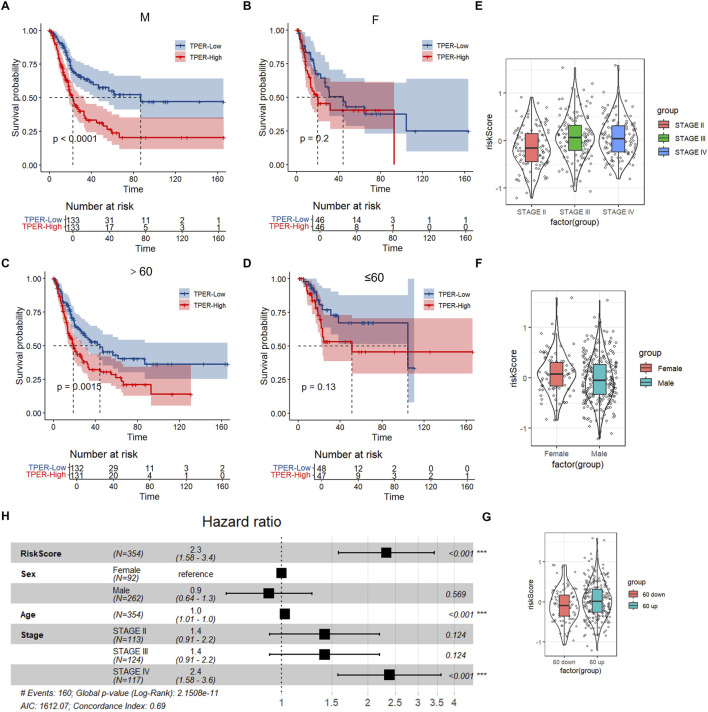
Subgroup survival analysis and multivariate Cox regression. Forest plot of Cox regression analysis and prediction in the study population. **(A)** Kaplan-Meier curve for overall survival (OS) in male patients stratified by the RiskScore (TPER-Low vs. TPER-High), with a P-value <0.0001. **(B)** Kaplan - Meier curve for OS in female patients stratified by the RiskScore (TPER-Low vs. TPER-High), with a P-value of 0.2. **(C)** Kaplan - Meier curve for OS in patients aged >60 years stratified by the RiskScore (TPER-Low vs. TPER-High), with a P-value of 0.0015. **(D)** Kaplan-Meier curve for OS in patients aged ≤60 years stratified by the RiskScore (TPER-Low vs. TPER-High), with a P-value of 0.13. **(E)** Violin plot showing the distribution of risk scores across different tumour stages (Stage II, Stage III, Stage IV). **(F)** Violin plot showing the distribution of risk scores between female and male patients. **(G)** Violin plot showing the distribution of risk scores between patients with different age-related groups (60 down vs. 60 up). **(H)** Forest plot of multivariate Cox regression analysis showing the hazard ratios (HR) and 95% confidence intervals for risk score, sex, age, and tumour stage, with global P-value from Log-Rank test and concordance index.

We developed a nomogram model that integrates clinical and genetic factors for the diagnosis of bladder cancer. The model was constructed using four gene signatures and clinical characteristics, including age, sex, and disease stage. Notably, our results showed that the risk score was the most effective among all the variables tested, including age, sex, disease stage, and the four-gene signature (Figures 8A–F,H). The four-gene signature showed that the prognosis for the high-risk score group was significantly worse than that of the low-risk score group across different age, grade, and gender groups (p < 0.05). This indicates that the high-risk score group has a poorer prognosis than the low-risk score group, demonstrating that the model has strong predictive power.

These findings suggest that the risk score could serve as a valuable prognostic marker for the disease, aiding in the personalised treatment of patients. In summary, our nomogram provides a valuable tool for predicting disease risk, highlighting the potential of integrating clinical and genetic factors to improve prognostic accuracy. Overall, these data demonstrate that our four-gene signature model has strong clinical predictive performance.

The clinical independence of the four-gene signature in the TCGA dataset was confirmed through multivariate Cox regression analysis, which revealed that the risk score was significantly associated with survival (Figure 8G).

### *OAS1* inhibits proliferation, migration, and invasion of bladder cancer

**FIGURE 9:**
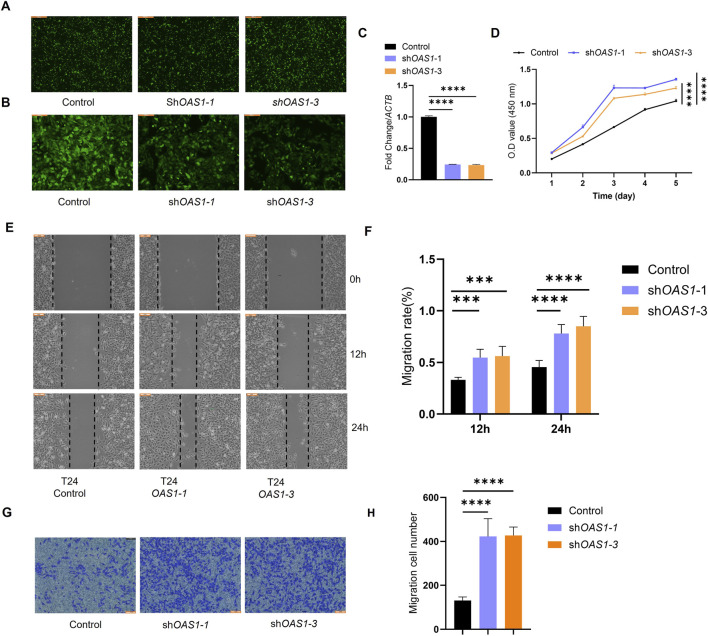
The role of OAS1 in the proliferation, migration and invasion of bladder cancer cells. **(A)** Fluorescence microscopy images showing the transfection efficiency of OAS1-specific shRNAs (shOAS1-1, shOAS1-3) and control in bladder cancer cells (scale bars, 100 μm). **(B)** Higher magnification fluorescence microscopy images of the Infected cells, confirming the knockdown efficiency (scale bars, 100 μm). **(C)** Statistical analysis was performed using ordinary one-way ANOVA followed by Dunnett’s multiple comparisons test. Data are presented as mean ± SEM (n = 5). Comparisons were made between the control group and each knockdown group (P < 0.0001). **(D)** Cell proliferation was measured by CCK-8 assay in bladder cancer cells transfected with two independent shRNAs targeting OAS1 (shOAS1-1 and shOAS1-3) and a non-targeting control (Control) over 5 days. Data are presented as mean ± SD from at least three independent experiments. **(E)** The effect of knockdown of designated genes in T24 cells on cell migration was assessed using a wound-healing assay. **(F)** Quantitative analysis of wound healing assay shown in. **(E)** Statistical analysis was performed using ordinary one-way ANOVA followed by Dunnett’s multiple comparisons test. **(G)** The effect of OAS1 knockdown on the invasive ability of T24 cells was detected by Transwell assay (scale bars, 1 mm). **(H)** Quantitative analysis of wound healing assay shown in. **(G)** Statistical analysis was performed using ordinary one-way ANOVA followed by Dunnett’s multiple comparisons test.

To investigate the functional roles of the four selected genes in bladder cancer, we focused on *OAS1*, which has the highest expression. We knocked down *OAS1* using technology to assess its effects on bladder cancer, specifically its influence on the proliferation, migration, and invasion abilities of bladder cancer cell lines (T24 cells) (Figures 9A–H).

293 after transfection, the fluorescence results showed uniform (Figure 9A). Fluorescence 72 h after infection with T24 and Puromycin showed that the control had the strongest fluorescence, followed by *OAS1*-1 and *OAS1*-3 (Figure 9B). qPCR results showed that knockdown of *OAS1*-1 and *OAS1*-3 was significant compared with the control (Figure 9C).

In CCK-8 experiments, we observed that OAS1 knockdown significantly promoted T24 cell proliferation compared with the control group (Figure 9D). This suggests that this gene plays an essential role in inhibiting cell growth. To evaluate the effect of gene knockdown on cell migration, we performed a scratch healing assay. The results showed that knockdown of OAS1 significantly promoted cell migration compared with the control group (Figures 9E,F). The Transwell assay was used to assess cell invasion ability. Compared with the control group, OAS1 knockdown led to a significant increase in cell invasiveness, highlighting its key role in inhibiting cell invasion (Figures 9G,H).

**FIGURE 10:**
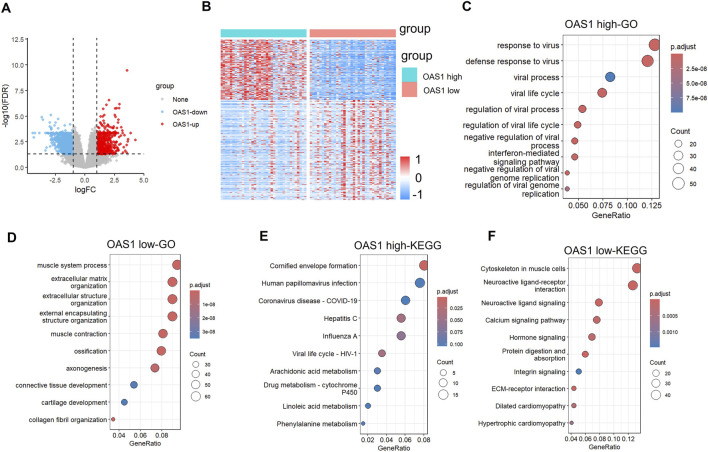
Differential gene expression analysis and functional annotation. **(A)** Volcano plot of differentially expressed genes in two groups. **(B)** Heatmap of differentially expressed genes in two groups. **(C)** Biological process (BP) annotation map of differentially upregulated genes. **(D)** (Biological process) BP annotation map of differentially downregulated genes. **(E)** KEGG annotation map of differentially upregulated genes. **(F)** KEGG annotation of differentially downregulated genes.

To investigate the molecular mechanism through which OAS1 exerts its function, a differential gene expression analysis was conducted between the OAS1 high-expression group and the low-expression group using strict criteria (|log2FC| > 1 and FDR <0.01). We performed an exploratory analysis using the publicly available TCGA-BLCA cohort. We stratified the cohort based on *OAS1* expression levels, selecting the top 10% (n = 36) as the high-expression group and the bottom 10% (n = 36) as the low-expression group. This analysis identified 739 genes that were significantly dysregulated, including 277 up-regulated and 462 downregulated genes in the high-expression group compared to the low-expression group. Notably, most of the DEGs showed higher expression in the low-expression group (Figure 10A). Unsupervised hierarchical clustering of these DEGs demonstrated clear expression patterns that separated the two groups, further confirming their molecular differences (Figure 10B). GO and KEGG enrichment analysis (Figures 10C–F) of differentially expressed genes in BLCA revealed that up-regulated genes were significantly enriched in viral infection-related pathways (such as HPV, COVID-19, HIV-1 viral life cycle, and immune response), metabolic processes (e.g., arachidonic acid metabolism and cytochrome P450-mediated drug metabolism), as well as cornified envelope formation. Meanwhile, down-regulated genes were primarily enriched in muscle system processes, extracellular matrix organization, cytoskeletal regulation, neuroactive ligand-receptor interaction, calcium signaling pathway, and cardiomyopathy-related pathways. These findings suggest that in bladder cancer, immune–virus interactions and metabolic reprogramming may promote tumor progression. At the same time, the suppression of muscle structure and cell adhesion-related pathways may be associated with the tumor’s invasive phenotype.

## Discussion

Patients with bladder cancer often do not show clinical symptoms in the early stages of the disease. Because of the high rates of morbidity and mortality, treating these patients remains a significant global public health challenge. Given the substantial heterogeneity in the natural course and prognosis of bladder cancer, identifying reliable molecular markers that can accurately stratify patients and predict clinical outcomes is crucial for management. This is crucial for informing clinical decisions and enhancing patient survival rates. Although previous studies have proposed prognostic models for gliomas, there has been limited research specifically focused on the bladder cancer subgroup for prognostic assessment based on tumor progression of epithelial cells. Our study aimed to reveal molecular subtypes of bladder cancer with different prognostic outcomes by analysing gene expression patterns associated with TPECs. Additionally, we identified and validated a novel four-gene marker as an independent prognostic indicator for bladder cancer. Implementing such reliable prognostic markers in clinical practice can aid in patient consultation, treatment planning, and screening for clinical trials, ultimately enhancing the prognosis of bladder cancer patients.

Many studies have highlighted several tumour prognostic models. However, few studies have focused on screening prognostic markers for bladder cancer based on tumour progression epithelial-associated genes. In the present study, we constructed a novel and highly reliable four-gene marker (*OAS1*, *AOC2*, *HOXB5*, and *HSH2D*) for predicting the prognosis of bladder cancer based on TPECs-related genes. We validated the marker in a separate database.

*OAS1*, a member of the OAS family, has been shown to be a family of interferon-induced enzyme proteins (Sarkar et al., 1999). A pan-cancer analysis revealed that members of the OAS gene family exhibit upregulated expression in most tumors, with a particularly significant upregulation in Gastric cancer (GC) (Zhang et al., 2025). Furthermore, overexpression of OAS1 affects survival in lung adenocarcinoma (LUAD) patients and immune cell infiltration, suggesting it may serve as a potential prognostic marker for LUAD (Wang et al., 2024). The OAS family is involved in various intracellular functions, including the induction of apoptosis, enhancement of IFN-α signalling response, gene regulation, regulation of immune cell receptors, and autophagy (Justesen et al., 2024). In addition, OAS1 has been shown to be associated with various subcellular components, including mitochondria, nuclei, and rough/smooth microsomes (Hovanessian et al., 1987; Chebath et al., 1987). Studies have found that OAS1 is highly expressed in many tumours (Kondratova et al., 2020). And its high expression is associated with poor prognosis of breast cancer (Zhang and Yu, 2020). Its prognostic value in breast cancer clinical intervention has been demonstrated, as it is a gene that plays a vital role in antiviral immunity. Compared with normal pancreatic tissue, OAS1 is highly expressed in pancreatic cancer tissue, and high expression of OAS1 is associated with a low overall survival rate (Lu et al., 2022).OAS1 may be involved in Trastuzumab-resistant gastric cancer (Yu et al., 2018). It has been confirmed that downregulation of OAS1 expression leads to a decrease in cell motility *in vitro* (Marino et al., 2014). Furthermore, OAS1 may be regulated by 17β-estradiol (E2) and play a crucial role in inducing apoptosis in cancer cells (Smekens et al., 1986; Silvestro et al., 1989). It is closely related to the occurrence and development of gastric cancer (Yu et al., 2018), breast cancer (Lu et al., 2021), lung adenocarcinoma (Song et al., 2020), and bladder cancer (Qiu et al., 2020). Although OAS1 is associated with poor prognosis in certain cancers (such as breast cancer), our data in bladder cancer indicate that high expression of OAS1 correlates with a better prognosis, suggesting that its role may be cancer type-specific and warrants further investigation.

AOC2 is a member of the copper-binding amine oxidase superfamily (Zhang et al., 2003). *AOC2* shares sequence identity with the human kidney Diamine oxidase gene (*KAO*, *AOC1*) and vascular adhesion protein-1 (VAP-1, AOC3). There are currently no studies of it in tumours. HOXB5, a member of the Hox gene cluster, plays not only a crucial role in development (Uversky et al., 2011; Kam and Lui, 2015) but is also strongly associated with multiple cancers (Hong et al., 2015; Zhang et al., 2018; Tucci et al., 2011; Lee et al., 2015). For example, Zhang et al. reported that HOXB5 expression was significantly increased in non-small-cell lung carcinoma tissues compared with normal tissues, and knockdown of HOXB5 inhibited cell proliferation and motility (Zhang et al., 2018). Studies have shown that HOXB5 is frequently overexpressed in bladder cancer tissues and cell lines, and the inhibition of HOXB5 can suppress the carcinogenic function of cancer cells (Yin et al., 2025; Chiariotti et al., 2012). HSH2D, previous studies have shown that it plays a crucial role in the resistance of T-cell acute lymphoblastic leukaemia (T-ALL) to methotrexate (MTX), providing a potential research target for studying T-ALL resistance (Wang and Xiong, 2020). In addition, it is also a marker of poor prognosis, as it is highly upregulated in proliferating cells that survive doxorubicin, and its expression is associated with U-ISGF3-related genes (Trzaskoma et al., 2024). However, there is no study on bladder cancer.

## Conclusion

In our study, we constructed a subtype of bladder cancer model based on TPECs-related genes. Using the DEGs identified in the BLCA subtype, we built a prognostic risk model comprising four genes (OAS1, AOC2, HSH2D, and HOXB5) and validated it using the E-MTAB-4321 arrayExpress dataset. Although our results reveal interesting phenomena and novel genetic signatures, they also have limitations. Although we have been validated effectively using an external database, additional data from multiple platforms is needed to confirm the validity of our model further. The four genes identified in this study deserve in-depth exploration through cell experiments and animal studies to elucidate their roles in TPECs and BLCA, thus laying the foundation for their potential clinical application in BLCA.

## Data Availability

The datasets presented in this study can be found in online repositories. The names of the repository/repositories and accession number(s) can be found in the article/Supplementary Material.
